# Rodlet Cell Morpho–Numerical Alterations as Key Biomarkers of Fish Responses to Toxicants and Environmental Stressors

**DOI:** 10.3390/toxics12110832

**Published:** 2024-11-20

**Authors:** Maurizio Manera

**Affiliations:** Department of Biosciences, Food and Environmental Technologies, University of Teramo, St. R. Balzarini 1, 64100 Teramo, Italy; mmanera@unite.it

**Keywords:** teleosts, immune response, environmental monitoring, biomarkers, toxicology, pollutants, aquatic ecosystems, pathophysiology, One Health

## Abstract

Rodlet cells (RCs) are specialised immune cells found in teleost fish, recognised for their unique morphology and potential roles in both immune responses and environmental adaptation. Herein, current knowledge on RCs is reviewed, focussing on their responsiveness to toxicants and environmental stressors. The historical context of RC research is discussed, including key milestones in the identification and characterisation of these cells. Recent studies highlight RCs’ quantitative and qualitative changes in response to various pollutants, such as heavy metals, organic chemicals, and microplastics, underscoring their utility as biomarkers for environmental monitoring and assessment of ecological health. The underlying mechanisms that govern RC responses are explored, noting the limited research available at the molecular level, which hampers a comprehensive understanding of their functionality. Despite this, the consistent patterns of RC responses position them as valuable indicators of environmental health within the One Health framework, linking aquatic ecosystem integrity to broader human and animal health concerns. Additionally, the potential equivalence of RCs in other vertebrates is examined, which may provide insights into their evolutionary significance and functional roles across different species. The urgent need for further research is emphasised to enhance the understanding of RC biology and its applications in toxicology and environmental pathology.

## 1. Introduction

Fish hold a pivotal position in scientific research as vital representatives of vertebrates, offering profound insights into diverse biological systems. Among them, the zebrafish (*Danio rerio*) stands as a quintessential model organism, propelling advancements in genetics, diseases, and drug development owing to its fully sequenced genome [1,2,3,4]. Despite this acclaim, the presence of rodlet cells (RCs) in not only zebrafish but also in other fish species remains a tantalising yet underexplored aspect [5,6].

These enigmatic RCs, unique to teleosts, inhabit strategic locations in mucosal surfaces like the skin, gills, intestine, and other internal tissues [5,6]. Characterised by distinctive rod-shaped granules, these specialised cells appear to play a significant role in the fish’s immune defence against pathogens [5,6,7]. Delving into the realm of RCs within teleosts offers a promising avenue of research, unveiling unique immunological strategies evolved in fish and broader evolutionary adaptations. Understanding the mechanisms governing these specialised cells not only enriches our comprehension of fish biology but also holds substantial potential for biomedical applications [5,6,8,9].

Historically, morphological assessment has been fundamental in deciphering cellular functions, relying on structural analysis to unravel aspects of various cell types [10]. This approach remains pivotal, especially in studying RCs, given the early stage of biomolecular exploration in comprehending these cells [5,8]. While morphological assessment serves as an initial foothold in understanding cellular functions, further advancements into the biomolecular realm are essential to unlocking the complete functional panorama of RCs [5].

The first comprehensive review on RCs [5] followed the “First International Rodlet Cell Workshop” held in Ferrara, Italy, in June 2001, where the endogenous origin of RCs was confirmed by the attending researchers. This workshop marked a pivotal moment in RC research, clarifying ambiguities surrounding their nature and function. Since then, the understanding of RCs has evolved considerably, particularly with recent updates [6] focussing on their role as immune effector cells, especially in defence against parasitic organisms. These updates underscore RCs’ complex behaviour and involvement in immune responses, an area that has drawn increasing interest. Furthermore, a recent paper critically compared the structure of RCs and Apicomplexan protozoa, reinforcing the argument against a parasitic origin for these enigmatic cells [11], thereby adding a new dimension to the ongoing debate regarding their true biological nature.

This review synthesises the latest insights into RCs of teleosts, particularly their responses to toxicants and environmental stressors, within a broader pathophysiological framework. For the purposes of this review, environmental stressors are defined as any variations in environmental variables, whether naturally or artificially occurring, that compel an organism to respond in order to maintain homeostasis [12]. A critical re-evaluation of the historical background of RC research is presented, identifying key milestones, misinterpretations, and controversies that have shaped current understanding. Additionally, the diagnostic features of RCs, including their cellular morphology and potential functions, are examined, with a particular focus on findings from morphological biological disciplines, drawing upon both classical studies and recent advances. By integrating insights from these morphological approaches, this review aims to provide a comprehensive overview of rodlet cell biology while highlighting knowledge gaps that warrant further investigation.

## 2. Key Milestones in the Timeline of RC Discovery and Understanding

The identification of RCs dates back to 1892 when the French protozoologist Thélohan initially described them as “indeterminate sporozoans” [13]. Despite their prevalence in teleosts, these cells might have been observed before but went unrecognised due to their distinctive characteristics. This oversight could be attributed to “inattentional blindness”, a psychological phenomenon where an individual fails to notice a clearly visible object or event because their attention is focused elsewhere [14]. It is plausible that histologists of that era noticed these cells in fish tissue but dismissed them due to their unique features, considering them foreign entities or potential parasites outside their scientific focus. Interestingly, the first detailed description of RCs came from a parasitologist, likely because rodlet cells superficially resembled sporozoans when viewed under a light microscope [5,15]. Furthermore, the limited number of studies on RCs in subsequent years, despite increasing interest in fish as valuable models in biomedical sciences, suggests a persistent potential for inattentional blindness over time.

Another contributing factor to the misrecognition/misinterpretation of RCs could be the linguistic barrier. The initial articles describing RCs were written in French in 1892 by Thélohan [13], in 1895 by Laguesse [16], and in German in 1906 by Plehn [17]. Laguesse and Plehn realised they referred to the same cells and engaged in a written debate in the German scientific journal *Anatomischer Anzeiger*, differing on their perceived parasitic and glandular nature [18,19]. When the English researcher Duthie [20] described and documented cells recalling RCs in Labridae and Triglidae intestines in 1939, he failed to recognise them, despite RCs being previously described as parasites [13,16,18] and fish cells [17,19]. He rather considered them granulocytes altered and discharged during their passage through the mucous membrane of the intestinal wall [20]. In 1949, Al-Hussaini [21,22], an Egyptian researcher at Sheffield, UK, did not recognise the already described RCs and referred to them as “pear-shaped” cells, associating them with a stage of goblet cells. In 1951, Catton [23], another English researcher, described RCs in roach, trout, and perch as “discharging coarse granulocytes”, disregarding previous RC references apart from Duthie’s [20]. Interestingly, Catton [23] referenced a private communication from Al-Hussaini concerning the possible lipoid nature of the granules in these cells yet did not acknowledge Al-Hussaini’s characterisation of them as “pear-shaped” cells.

Though the term “rodlet cell” was first introduced by Bullock in 1963 [24] and an article in Japanese [25] considered “pear-shaped” cells synonymous with RCs, asserting their recognition as well-established, other authors from English-speaking countries [26,27] did not clearly acknowledge “pear-shaped” cells as RCs. This indicates a persistent misrecognition/misinterpretation, possibly influenced by a linguistic barrier hindering access to literature in languages other than English and/or understanding scientific disciplines beyond their own. Interestingly, in 1967, Westerman and Wilson [28], two Australian researchers, documented the ultrastructure of RCs in the olfactory mucosa of *Carassius auratus*, referring to them as “foliaceous cells” and suggesting a possible receptor function without acknowledging any prior research on RCs. Similarly, in 1971, Fearnhead and Fabian [29], two South African researchers, described the ultrastructure of RCs in the gills of *Monodactylus argenteus*, referring to them as “rod cells” and speculating about their potential role in osmoregulation, again without referencing earlier studies on RCs. A further consequence of linguistic barriers may be seen in the relative neglect and misinterpretation of articles published in Russian journals [30,31,32,33,34,35,36,37]. Despite their informative content, these works often go under-cited, raising the question of whether citations, where they occur, are based on a thorough interpretation of the original Russian texts, merely on the English abstracts provided by the authors, or on secondary citations.

In a notable example of potential misinterpretation, Egyptian researchers [38,39] purportedly described the presence of RCs in two bird species from a vertebrate class distinct from teleosts. Despite approximately five years having elapsed since these descriptions, no subsequent studies, including those by the original authors, have reported RCs in the same or other avian species. As highlighted by Dezfuli et al. [6], it is improbable that RCs would be present in a vertebrate class as phylogenetically distant from teleosts as birds, especially when absent in more closely related vertebrate classes, such as non-teleost fish.

Here is a brief timeline highlighting the key points:

1. Discovery (late 1800s–mid-1900s): RCs were initially observed and characterised during this period. Early researchers identified their distinctive rod-shaped granules and proposed various hypotheses regarding their nature, speculating that they might be parasites [13,16,18], glandular cells (referred to as “Stäbchendrüsenzellen” by German fish pathologist Marianne Plehn) [17,19], altered, discharged granulocytes [20,23] or a stage of goblet cells [21,22].
2. Further characterisation (mid-1900s–late 1900s): During this period, scientists conducted comprehensive investigations into RCs, employing advanced techniques such as transmission electron microscopy. The prevailing consensus leaned towards an endogenous origin, positing that these cells originated from the fish itself [23,24,26,40,41,42,43,44,45,46,47,48,49]. However, the possibility of a parasitic origin was not entirely discarded by some authors [50,51,52,53,54,55].
3. Pathogen interaction and immune function (mid-1900s–present): During this period, extensive research was focused on elucidating the interactions between RCs and various pathogens, encompassing micro- and macroparasites [5,6,7,9,24,43,46,56,57,58,59,60,61,62,63,64,65,66,67,68,69,70,71,72,73,74]. Additionally, investigations explored interactions with bacteria [75,76] and, in a single isolated reference, viruses [77]. These studies significantly advanced our understanding of immune responses in fish. The interaction between RCs and pathogens emerged as a crucial aspect within the realm of fish immune responses [5,6,7,9,73,78].
4. Response to toxicants and environmental stressors (mid-1900s–present): RCs have demonstrated responsiveness to various toxicants, including heavy metals, organic pollutants, native and polluted PVC pellets (microplastics), and stressful environmental conditions (such as salinity stress, wounds, and overcrowding) [8,27,29,30,31,33,34,79,80,81,82,83,84,85,86,87,88,89,90,91,92,93,94,95,96,97,98]. Their response involves changes in their number, distribution, morphology, and degranulation, rendering them valuable as generalist biomarkers indicative of environmental contamination and stressors [5,8,83,84,86,90,94,97,99,100,101].
5. Biomolecular research (early 2000s–present): The current state of biomolecular tools, particularly those derived from omics disciplines, for investigating RCs remains relatively underdeveloped. Comprehensive molecular analyses specifically targeting RCs have not been fully realised. Instead, methods such as immunofluorescence and immunohistochemistry have predominantly been employed to delve into the intricate details of rodlet cell biology. These methodologies have been instrumental in examining various aspects of RCs’ structural and functional properties, including investigations into the cytoskeletal framework [11,102,103,104,105,106]; cytokine secretion patterns (e.g., TNF, IL-1β) [107,108,109]; key antigen expression (e.g., TLR, S100) [90,105,108,109,110,111]; secretion patterns of molecules like lysozyme, piscidine, melanocyte-stimulating hormone, and i-NOS [73,75,88,112,113]. Additionally, studies have explored the presence of stem cell markers (e.g., CD117, CD34), matrix metalloproteinase-9, and vascular endothelial growth factor (VEGF) [114], as well as calreticulin [115], alpha-7 nicotinic receptor, NF-κB, ionised calcium-binding adapter molecule 1 (IBA1), oligodendrocyte transcription factor 2 (OLIG2) [109,116], G-protein alpha subunit [117], CD68, nuclear factor erythroid 2-related factor 2 (NRF2), transcription factor SOX9, nicotinic acetylcholine R alpha 7 and myostatin [109]. The use of advanced molecular techniques is pivotal to uncovering the complete molecular landscape of RCs and gaining deeper insights into their functional roles within vertebrate biology. Consequently, ongoing advancements in molecular techniques are crucial for unlocking a more profound understanding of the intricate molecular mechanisms underlying RC biology [5,118].

## 3. Overview of Rodlet Cell Morphology

**Figure 1:**
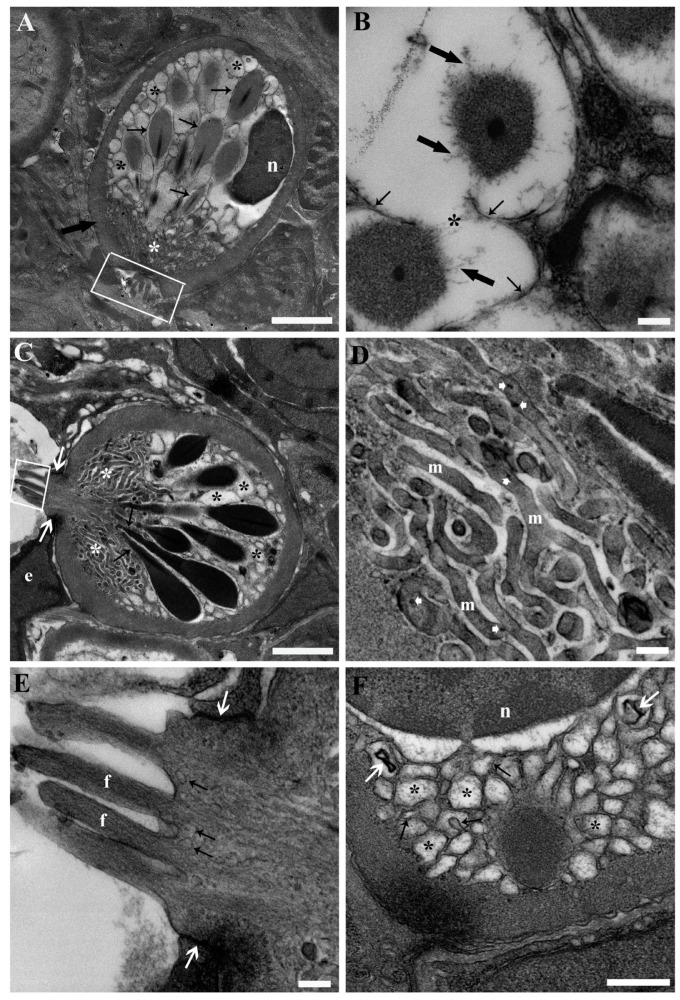
The plate illustrates the most distinctive diagnostic features of rodlet cells (RCs), highlighting their unique morphological characteristics. (**A**) A typical mature RC is characterised by a polarised, pear-shaped morphology, with an oval nucleus (n) located at the basal pole and prominent villous-like projections (rectangle) at the apical pole. Just beneath the cell membrane, a fibrillar sub-plasmalemmal layer is visible (thick arrow). The cytoplasm is distinctly vesicular (black asterisks) and contains rod-like granules, or rodlets (thin arrows), formed by an amorphous rodlet sac enclosing an electron-dense core that converges towards the apical pole, where numerous extremely thin, elongated, and branched mitochondria are abundant (white asterisk). Scale bar = 2 µm. (**B**) Membranes of neighbouring rodlets can fuse (asterisk) in certain RCs, along with the dissolution of rodlet sacs (thick arrows) and detachment of surrounding membranes (thin arrows), suggesting the occurrence of compound exocytosis. Scale bar = 200 nm. (**C**) A RC extending towards a sinusoid and projecting villous structures into its lumen (rectangle) is visible, accompanied by junctional complexes (white arrows) connecting to the sinusoidal endothelial cells (e). A mitochondrial network is apparent at the cell apex (white asterisks), with rodlets converging towards this region (arrows) and vesiculated cytoplasmic remnants (black asterisks) also evident. Scale bar = 2 µm. (**D**) The apical mitochondrial network is composed of unique, elongated, slender, and branched mitochondria (m), displaying the characteristic matrix granules (arrowheads). Scale bar = 200 nm. (**E**) The crown-shaped, villous projections containing a fibrillar core (f), which extends into the upper cytoplasmic part of the cell, along with vesicles (arrows) at their base, are visible. Junctional complexes (white arrows) between the RC and the endothelial cells are clearly visible. Scale bar = 200 nm. (**F**) Numerous vesiculations (asterisks) are present in the cytoplasmic area surrounding the nucleus (n), some of which show protrusions (black arrows) extending into adjacent vesicles. Additionally, lamellar bodies are easily identifiable (white arrows). Scale bar = 500 nm. Transmission electron micrographs of RCs from the kidneys of common carp (*Cyprinus carpio*), reproduced, under an open access Creative Commons CC BY 4.0 license, from Manera, M.; Castaldelli, G.; Giari, L. Perfluorooctanoic acid promotes recruitment and exocytosis of rodlet cells in the renal hematopoietic tissue of common carp [8].

While slight morphological variations may occur among species, the recognised mature stage of RCs exhibits distinct key features observable under light microscopy [5,6,8,119] and, most effectively, with transmission electron microscopy (Figure 1A–F) [8,11,45,48,96,120,121]. These include a piriform or elliptical shape, allowing for the visualisation of major and minor axes (Figure 1A,C). Notably, the cell demonstrates a significant thickening of its peripheral cytoplasm, giving the false appearance of a “capsule” (Figure 1A,C,F). At one end, known as the basal pole, a conspicuously large nucleus is evident (Figure 1A,F). The defining characteristic of these cells is the presence of elongated rod-shaped granules extending from the supranuclear area towards the opposite pole, referred to as the apical pole (Figure 1A,C). Typically, this pole is orientated towards epithelial surfaces, where RC projections may extend into the surrounding space, depending on the anatomical location (Figure 1A,C,E). Additionally, the residual cytoplasm appears clear and vesiculated (Figure 1A,C,F) [5,6,8,119].

The study focussing on RCs’ dimensions stands as a relatively unexplored area, except for a significant investigation evaluating interspecific and intraspecific (specifically, inter-organ) variations in RCs’ axes [119]. Notably, mean values for major and minor axes ranged between 10.5 µm (kidney tubules of *Gasterosteus aculeatus aculeatus*) and 19.2 µm (bile ducts of *Carassius auratus auratus*) and 7.1 µm (bile ducts of *Dicentrarchus labrax*) and 10.9 µm (intestine of *Oncorhynchus mykiss*), respectively, varying across species and anatomical sites [119]. This comprehensive study not only unveiled differences among species but also revealed variability within species, partly associated with variations in the heights of the epithelial cells where RCs are situated [119]. Consequently, this observation led to the hypothesis proposing the existence of various morphotypes of RCs [119], as previously suggested [41,49,84]. However, despite approximately fifteen years passing since the publication of that study, the lack of reliable biomolecular tools capable of confirming or refuting this hypothesis remains a challenge.

At the ultrastructural level, the “capsule” of the RC appears to be composed of a fibrillar layer containing contractile filaments, which line the cytoplasmic side of the plasmalemma (Figure 1A,C,F). This fibrillar layer is reduced or absent at the apical pole of the RC, where the tips of the rodlets converge (Figure 1A,C,E). The rodlets exhibit an outer amorphous structure, referred to as the rodlet sac, and a more electron-dense core (Figure 1A–C). The Golgi apparatus is visible in the paranuclear area and is associated with numerous vesicles, while the endoplasmic reticulum is virtually absent or negligible in mature RCs. Scattered free ribosomes are present throughout the cytoplasm, especially notable adjacent to the inner surface of the fibrillar layer. At the apical pole, there is a characteristic accumulation of thin, elongated mitochondria (Figure 1A,C,D) [8,11,45,48,96,120,121].

Histochemistry has been the principal method for characterising RCs, particularly in analysing the chemical composition of their unique rod-shaped granules [27,122]. However, the comprehensive characterisation of rodlet content remains relatively underexplored. Leino [47] was the first to thoroughly detail the histochemistry and cytochemistry of rodlets, demonstrating histochemical positivity for carbohydrates and proteins in the rodlet sac, while no reactions were observed to lipid-specific or nucleic acid stains. In contrast, the rodlet core stained positively for proteins but did not react with carbohydrates, nucleic acids, or lipids. Electron microscopy showed the rodlet sac’s resistance to protease digestion and positive staining with periodic acid-silver methenamine, indicating a significant glycoprotein presence. The rodlet core, susceptible to protease digestion and negative for silver methenamine staining, was predominantly composed of proteins [47]. Vickers [27] had reported a lipid component in rodlets, previously proposed by Catton [23], although Leino [47] did not confirm this in his findings. More recently, Abd-Elhafeez and Soliman [123] reported similar observations of lipid components. Additionally, Iger and Abraham [97] identified enzymes in mature RCs from carp and trout, including alkaline phosphatase in the rodlet sac and peroxidase in the core, through immunogold labelling techniques. Bosi et al. [75] further observed immunostaining positivity for inducible nitric oxide synthase (i-NOS) and lysozyme in RCs of carp and eel, along with histochemical reactivity to lectins such as ConA, SNA, WGA, and DBA (the latter two only in carp). Interestingly, WGA positivity in rodlets had been previously reported by Imagawa et al. [124] in carp.

RCs are capable of discharging their rodlets, a process predominantly reported to occur through a holocrine mechanism, where the plasmalemma ruptures abruptly, and the entire cytoplasm is expelled from the cell, potentially due to the active contraction of the fibrillar capsule [11,48,102]. However, Hawkins [96] observed the discharge of individual rodlets via a merocrine mechanism, where the integrity of the plasmalemma is maintained through the fusion of the plasma and rodlet membranes. Additionally, some authors have suggested the occurrence of an apocrine mechanism [111,125]. More recently, piecemeal and compound exocytosis have also been reported in RCs [8]. The secretory products of RCs are believed to possess antibiotic properties against pathogens, thereby facilitating tissue healing [49,97]. Notably, immunopositivity to the biocidal peptide piscidin was observed in RCs of a single specimen of *Oreochromis niloticus* [113].

## 4. Possible Rodlet Cell Equivalent in Other Vertebrates

Understanding the potential equivalence of RCs in other vertebrates significantly enhances our comprehension of their nature and function. Current hypotheses have categorised RCs either as secretory cells or as a specific type of leukocyte resembling granulocytes. These hypotheses are derived from conflicting observations of RCs; their migratory behaviour and interaction with vascular districts support the leukocyte hypothesis, while their association with epithelia, alongside the presence of junctional complexes with epithelial cells, favours the secretory cell hypothesis [5,6,9,41,48,50,72,126]. This disparity in evidence has been previously highlighted [5,41].

Presently, there exists no definitive analogy between differentiated cells of other vertebrates and RCs. However, potential similarities with undifferentiated cells warrant attention, particularly concerning their migratory tendencies and formation of junctional complexes. Notably, the epithelial-to-mesenchymal transition (EMT) cell is a migratory cell capable of forming desmosomes and tight junctions with epithelial cells [127]. EMT is a biological process wherein epithelial cells lose polarity and adhesion, adopting mesenchymal traits such as increased motility and invasiveness [127]. EMT cells can revert to epithelial cells through mesenchymal-to-epithelial transition (MET), re-establishing desmosomes and tight junctions with surrounding epithelium [128]. These dynamic transformations, integral to EMT and MET, play pivotal roles in various physiological and pathological processes, including development, wound healing, and cancer [129]. Additionally, recent research has suggested an intermediate cell state (ICS) during the EMT transition, exhibiting mixed characteristics of epithelial and mesenchymal states [130]. Given these dynamic processes, RCs in teleosts may potentially represent a distinct cellular entity showing resemblances to transitional cellular phenomena observed in EMT, MET, and ICS, signifying a multifaceted and adaptable role within fish tissue. Notably, prior suggestions have proposed that RCs may embody a distinctive cell type in teleosts, displaying attributes common to both leukocytes and epithelial secretory cells [5]. Furthermore, recent findings have hinted at the presence of stemness properties within RCs, coupled with their migratory capabilities [114]. Additionally, a potential mesenchymal-like origin has been suggested for these cells [123]. Notably, certain mesenchymal-derived cells in fish, such as epithelioid cells (macrophage-transformed cells typical of granulomata) [131] and fibroblast-like reticular cells within macrophage aggregates [132], exhibit desmosomes.

Nevertheless, a more conservative hypothesis should be considered that mature RCs lack the capacity to migrate through tissues. This theory posits that mature RCs have a somewhat “rigid” and undeformable structure due to their fibrous capsule and the absence of lamellipodia, thus explaining the lack of migratory activity [11,103,118]. Instead, this hypothesis suggests that immature RC precursors may be responsible for the presumed migratory properties of RCs [103].

While lamellipodia have not been detected in mature RCs [11,103], villous-like projections have been widely reported at the apex of RCs, particularly in regions facing the lumen of blood vessels, cavitary organs, and body surfaces [101,125,133,134,135]. The resemblance of these villous projections to T-cell microvilli has been noted, supporting the role of RCs in innate cellular immunity [8,136]. Iger and Abraham [98] referred to these structures as an “apical tuft”, and Montoro et al. [137] highlighted the similarity between many key features of RCs and those of tuft cells, which are chemosensory cells characterised by their apical tuft. This hypothesis was revisited by Willms and Foley [138], who commented on the absence of typical tuft cells in *Danio rerio*.

## 5. Rodlet Cells Response to Toxicants and Environmental Stressors

The study of RCs is closely linked to the investigations of their responses to various substances, including toxicants, miscellaneous chemicals, and environmental stressors. The first study reporting such a relationship dates back to 1962 [27], 62 years ago and 70 years after the initial description of RCs. This indicates that related research has spanned approximately half the duration of RC research, particularly coinciding with the adoption of ultrastructural, cytochemical, and immunocytochemical methods [92,96,97].

### 5.1. Bibliographic Overview

Table 1 summarises all known pertinent references on this topic, categorised by aetiological factors, primary outcomes, and the proposed mechanisms involved. It is important to note that, in certain instances (e.g., reference [97]), the same article is represented by multiple entries in the table, as it addresses exposure to different substances that are listed separately. With regard to the proposed mechanisms involved, this topic has been addressed only superficially to date, with few notable exceptions [86,88,89,101], and the underlying mechanisms have not yet been thoroughly studied at the molecular level. Notably, only approximately 33% of the table entries include a possible mechanism explaining the observed outcomes, and in only 60% of these cases (20% of the total entries), the outcomes are supported by appropriate statistical analyses. Overall, about 57% of the table entries incorporate statistical analyses to validate the reported outcomes. These are predominantly related to the quantitative variation of RCs following exposure to toxicants or environmental stressors. Only two table entries stand out for their focus on qualitative structural modifications; one relates to the PFOA-induced degranulation activity of RCs in a carp model, wherein texture analysis was employed to quantify these changes, with statistically significant differences assessed across experimental groups [99]. The other entry pertains to the position of RCs relative to the endothelium and their mode of discharge into the heart bulbar lumen following dexamethasone injection in a goldfish model [101]. Overall, irrespective of whether statistical analyses were performed, approximately 53% of the table entries refer to quantitative modifications in RCs, 10% to qualitative changes, and 37% to both quantitative and qualitative modifications. Additionally, only 30% of the listed entries rely exclusively on light microscopy, highlighting the substantial utilisation of transmission electron microscopy in the investigation of RCs’ responses to toxicants and environmental stressors. The crucial role of transmission electron microscopy in elucidating the mechanisms by which toxicants exert their effects has been previously highlighted [81,139,140,141].

### 5.2. Direct Versus Indirect Effects on Rodlet Cells

In reviewing the alterations and responses of RCs to toxicants and environmental stressors, it is essential to distinguish between those effects potentially induced directly at the cellular level and those resulting indirectly through integrated responses at the tissue, organ, or organismal levels. Unfortunately, to date, there is no published research specifically addressing the effects of toxicants and environmental stressors on isolated RCs. Furthermore, the research on the nature, function, and integration of RCs within piscine organisms is ongoing, though indirect evidence is emerging regarding their involvement in the immune system [5,6,7,9,11,73,78]. Notably, Schmachtenberg [118] studied isolated RCs from the olfactory rosettes of *Isacia conceptionis*, testing their responses to seawater and distilled water. The study concluded that RCs exhibited behaviour similar to other isolated cells, either shrinking or swelling depending on the osmolarity of the medium. Following this, DePasquale [103] proposed a scale explant culture method to facilitate the study of RCs in vivo using high-resolution Nomarski differential interference contrast microscopy. This research demonstrated immunopositivity to phosphotyrosine as a marker of signal transduction activity. The use of pervanadate, a tyrosine phosphatase inhibitor, was shown to trigger RC contraction, leading to the expulsion of their contents, which suggested that tyrosine kinases are involved in the contraction of the RC’s fibrous capsule [104]. Nevertheless, these previous studies should be regarded as physiological investigations, even though they may contribute indirectly to understanding some of the alterations and responses of RCs observed in toxicological studies.

The term “sentinel” in relation to RCs has been employed by some researchers [6,118] to emphasise their potential role as a first line of defence against infective agents rather than in the conventional immunological context. In immunology, a sentinel cell is typically defined as one that is equipped to detect and monitor pathogens or cellular damage via pattern recognition receptors (PRRs), such as Toll-like receptors (TLRs) and NOD-like receptors (NLRs). These receptors recognise pathogen-associated molecular patterns (PAMPs) and damage-associated molecular patterns (DAMPs), thereby initiating an immune response [143]. The expression of TLRs in RCs was reported by Alesci et al. in 2022 [105], and Manera et al. in 2023 [8] first suggested that the recruitment of RCs in fish exposed to perfluorooctanoic acid (PFOA) might be linked to PFOA-mediated TLR activation. Consequently, RCs should be considered as candidate sentinel cells, alternative to those traditionally recognised [8]. This perspective underscores both the immunotoxic effects of PFOA in fish and the potential role of RCs in responding directly to toxicants. However, the more conservative view that the activation/recruitment of RCs is triggered by tissue damage caused by toxicants or mediated by the activation of classical sentinel cells cannot yet be disregarded [8].

### 5.3. Numerical Variation and Recruitment of Rodlet Cells

The numerical variation of RCs in the reviewed studies predominantly shows an increase in their numbers, which is likely attributed to recruitment from immature precursors, as mature RCs do not exhibit PCNA immunoreactivity, a marker of mitotic activity [57]. However, some exceptions have been noted. In *Squalius cephalus*, either experimentally exposed to the herbicide Stam^®^ M-4 (Propanil) or subjected to manipulations intended to induce a stress response, a significant depletion of RCs in the bulbus arteriosus of the heart was observed [86]. It is important to note that *Squalius cephalus*, like other cyprinids, typically exhibits a high density of RCs in this region, especially immediately beneath the endothelium [5,9,45,50,86,101,135,144]. Similarly, species from other families, such as Poecilidae, have also been reported to have large quantities of RCs in the bulbus arteriosus. Interestingly, beyond the experimental findings in *Squalius cephalus* [86], a small field bioassay conducted with *Cyprinus carpio* demonstrated that fish residing in impacted channel waters showed a significant depletion of RCs in the bulbus arteriosus compared to fish in reference channel waters [145]. This observation aligns with the experimental data from *Squalius cephalus* and suggests that the bulbus arteriosus of the heart could serve as a marker organ for RCs response in species that typically host large numbers of RCs in this anatomical location. Another potential marker organ for RC response is the gastrointestinal tract of marine species, which rely on seawater drinking for water uptake and, consequently, may ingest toxicants dissolved in the water and/or adsorbed onto suspended particulate matter. These toxicants can directly impact the intestine and potentially recirculate through the enterohepatic pathway [100]. In fact, *Dicentrarchus labrax*, a marine euryhaline species, when experimentally exposed to inorganic (Cd, Hg) and organic (terbuthylazine) toxicants, exhibited the most pronounced relative increase in RC numbers in the intestine, correlating with the duration of exposure and toxicant concentration, compared to other tested organs (gills, kidney) [82,83,85].

### 5.4. Ultrastructural Changes of Rodlet Cells

With regard to the ultrastructural alterations widely reported in RCs following exposure to toxicants (refer to Table 1), these changes have generally been associated with toxic-degenerative effects [82,83,86]. However, similar ultrastructural alterations have also been observed in fish exposed to environmental stressors, such as osmotic shock [84], thereby challenging the hypothesis that such alterations should be regarded solely as the result of a direct toxic effect on RCs. For instance, the occurrence of myelin figures has been frequently reported in RCs as a response to toxicants. Dezfuli et al. [86] observed these structures in RCs of *Squalius cephalus* experimentally exposed to the herbicide Stam^®^ M-4 (Propanil) and suggested they may arise from membrane alterations due to toxicant exposure, inhibiting lysosomal digestion, as previously proposed [146]. The presence of such structures in fish subjected to environmental stressors [84] suggests that this phenomenon may represent a non-specific manifestation of cellular stress, characterised by increased membrane turnover, which at times may overwhelm normal cycling pathways [147]. Particularly noteworthy are the alterations observed in the rodlets themselves, which appear partially dissolute, especially at the periphery of the rodlet sac. Originally regarded as a non-specific, general degenerative response induced by toxicants or environmental stressors [84,86], these alterations have more recently been interpreted as forms of piecemeal and compound exocytosis, possibly mediated by TLR activation, specifically through direct toxicant-mediated mechanisms such as PFOA exposure [8]. Mature RCs typically exhibit small, elongated apical mitochondria, which are markedly different from the mitochondria of putatively immature RCs, the latter resembling the more commonly observed mitochondrial structure [96,148]. Hawkins [96] documented the presence of conventional mitochondria with cristae in fish exposed to toxicants, interpreting this as a possible cellular response. The occurrence of such classical mitochondria in RCs exposed to toxicants, potentially undergoing mitophagy (personal, unpublished observations), suggests that the presence of these traditionally structured mitochondria should not necessarily be interpreted as a direct response to toxicant exposure. Rather, it may indicate the recruitment of not fully mature RCs, which have yet to develop the characteristic thin, elongated, and branched mitochondria, devoid of cristae, which is typical of mature RCs.

### 5.5. Hormone-Mediated Responsiveness of Rodlet Cells

The potential responsiveness of RCs to hormones, particularly glucocorticoids, has been emphasised in several studies [88,89,101]. This has been demonstrated both through the induction of endogenous hormonal surges as part of the typical stress response [89] and through the exogenous administration of a synthetic glucocorticoid [101]. However, other researchers have suggested the involvement of stress-related factors distinct from cortisol [97,98]. Furthermore, the immunoreactivity of RCs to alpha-melanocyte-stimulating hormone (α-MSH) has been interpreted as indicative of an interaction between the immune and endocrine regulation of RCs [88]. These findings, once validated, may elucidate the link between RC responses across various levels of biological organisation, highlighting the integrative role of hormones in vertebrate physiology, particularly within the neuroendocrine–immune network [149,150], and may represent the pathophysiological basis of RC responses to environmental stressors and, in some instances, toxicants.

### 5.6. Generalist (Non-Specific) Response of Rodlet Cells and Ecotoxicological Relevance

Reviewing the table entries, it becomes evident that the quantitative and qualitative response of RCs should not be considered specific, as it remains substantially unaltered irrespective of the nature of the toxicant or environmental stressor. Nevertheless, this response appears sufficiently sensitive to support the use of RCs as generalist (non-specific) biomarkers, provided that appropriate controls are implemented to statistically account for other potential causes of the RCs’ response [5,79,90,100]. In other words, the RCs’ response should not be evaluated against reference values (which are unavailable) but rather against that of a control group, where the only significant difference is the exposure, whether natural or experimental, to known toxicants or environmental stressors. In cases where toxicants and environmental stressors are unknown, comparing RCs from fish in a pristine environment can serve as a generalist biomarker of fish health and, by extension, environmental health, in accordance with the One Health approach [151,152].

As previously noted, RCs’ responses may serve as generalist biomarkers indicative of environmental contamination and stressors [5,8,83,84,86,90,94,97,99,100,101], though Kramer et al. [153] questioned that RCs in *Fundulus heteroclitus* could be reliable biomarkers towards parasites and environmental stressors, and some criticism was also raised by Rideout et al. [154] commenting on the high RC density in the gonads of *Reinhardtius hippoglossoides*. Furthermore, the study by Nikolić et al. [155] in *Squalius cephalus* naturally exposed to mining effluents and municipal wastewater does not support the role of RCs as biomarkers, though it stresses the need for further study to speculate with confidence. Similarly, Muns-Pujadas et al. [156] showed that in *Merluccius merluccius*, the presence of RCs in the digestive tract is not associated with the abundance of anthropogenic items. Other authors stressed the need to consider possible sex variations when relying on RCs as biomarkers [79].

While the current review provides insights into RCs’ responses to various toxicants and stressors, significant knowledge gaps remain regarding the precise molecular mechanisms underlying these responses, particularly in ecotoxicological contexts. Bridging these gaps will be essential to enhance the application of RCs’ morpho–numerical alterations as reliable biomarkers in environmental toxicology, thereby enabling a more robust assessment of ecological health and organismal responses to complex pollutant mixtures. Notably, a foundational understanding of RC biology is crucial to confidently utilising RCs’ responses as biomarkers in ecotoxicological studies. Conversely, studies focusing on RCs’ reactions to toxicants and environmental stressors may also advance our knowledge of RC biology.

## 6. Conclusions

Rodlet cells exhibit a profound sensitivity to toxicants and environmental stressors, underscoring their potential as critical biomarkers in the fields of toxicology and environmental pathology. Their ability to respond dynamically to a wide array of harmful substances—ranging from heavy metals, organic chemicals, and microplastics to environmental stressors such as salinity shifts, overcrowding, and temperature fluctuations—positions them as versatile indicators of environmental health. These responses manifest through both quantitative changes, such as increases in RC numbers, and qualitative alterations, including modifications in their ultrastructure and secretory activity. Notably, RCs have demonstrated marked alterations in response to toxicants like cadmium, mercury, and PFOA, with specific structural changes linked to degenerative or adaptive processes within the cells. Such consistent reactions across various species and stressors highlight their utility in identifying and quantifying environmental impacts.

The potential of RCs extends beyond mere environmental monitoring, offering insights into the pathophysiological consequences of pollutant exposure. Their responses can be employed to assess sub-lethal toxic effects at the cellular level, providing an early warning system for ecological disturbances that may not yet manifest at the population level. This is particularly relevant in the context of toxicological studies, where the RC’s morphological and molecular alterations can serve as biomarkers to trace the impact of hazardous substances before more apparent signs of environmental or organismal damage occur. Importantly, RCs’ involvement in immune defence mechanisms, including their interaction with pathogens and their potential role in innate immunity, further amplifies their significance in fish health and disease resistance, contributing to a comprehensive understanding of the immune–toxicological nexus.

The application of RCs within the One Health framework is especially promising. This integrative approach recognises the interconnection between human, animal, and environmental health. RCs, as part of the fish’s immune system and their responsiveness to environmental changes, reflect the health status of aquatic ecosystems. As fish are often sentinel species, RCs provide an important link between aquatic environmental conditions and the broader implications for human and animal health. Their use in monitoring polluted waters, assessing the effects of contaminants on fish, and predicting potential health risks to humans who rely on aquatic resources aligns with the One Health perspective. By tracking changes in RCs in fish populations, scientists can gauge the level of environmental degradation and anticipate its cascading effects across species and ecosystems, contributing to early intervention strategies for ecosystem restoration and public health protection.

In summary, RCs hold substantial promise as biomarkers in toxicological and environmental pathology, offering a window into the complex interactions between organisms and their environment. Further research, particularly through biomolecular and omics-based approaches, is essential to fully elucidate the mechanisms by which RCs respond to toxicants and stressors. By advancing our understanding of these processes, RCs could be pivotal in promoting the One Health approach, reinforcing the need for integrated health strategies that safeguard both environmental sustainability and public health.

## Figures and Tables

**Table 1:** Bibliographic overview of alterations in rodlet cells (RCs) of fish exposed to toxicants and environmental stressors.

Etiological Category	Type of Toxicants/Environmental Stressors, Levels, and Duration of Exposure	Fish Species	Main Outcomes	Proposed Mechanisms Associated with Reported Effects	Adopted Methodology	Statistical Analysis	Reference
Inorganic chemicals	Co(NO_3_)_2_, CoSO_4_. After 8 days onwards of experimental exposure at 1.66 × 10^−3^ mol L^−1^ Co^2+^ and 3.33 × 10^−3^ mol L^−1^ Co^2+^. MnSO_4_. After 30 days of experimental exposure at an undefined concentration.	*Carassius auratus*	Unquantified increase in the number of RCs in the intestine and bile ducts.	Not provided.	Light microscopy on sections from paraffin-embedded samples.	Not provided.	[27]
	CdCl_2_ (from 10 to 100 mg L^−1^) for 48 h of experimental exposure.	*Leiostomus xanthurus*	Effect on RCs’ ultrastructure (qualitatively assessed) in the kidney.	Not provided.	Transmission electron microscopy on ultrathin sections from epoxy resin-embedded samples.	Not provided.	[96]
	NH_4_^+^ (0.5 and 2 mM) after 2 days of preconditioning in brackish water (26 ‰) for 5 days of experimental exposure.	*Pungitius pungitius*	Increase in the number and secretion activity of RCs in gills.	Not provided.	Light microscopy and transmission electron microscopy, respectively, on semithin and ultrathin sections from epoxy resin-embedded samples.	Not provided.	[33]
	Cd = 0.68 mg kg^−1^ in 200 g of pooled gill tissue from multiple fish naturally exposed to environmental pollutants. Cr = 3.7 mg kg^−1^ in 200 g of pooled gill tissue from multiple fish naturally exposed to environmental pollutants.	*Boops boops*	Increase in the number of RCs in gills.	Not provided.	Light microscopy on sections from paraffin-embedded samples.	Anova test on RCs’ number.	[90]
	Cd(NO_3_)_2_ (22, 500 µg L^−1^ Cd), Pb(CH_3_CO_2_)_2_ (0.5, 1 mg L^−1^ Pb), CuCl_2_ (100 µg L^−1^ Cu), up to 30 days of experimental exposure.	*Cyprinus carpio* *Oncorhynchus mykiss*	Appearance of RCs in the skin (semi-quantitatively assessed), particularly in fish exposed to Cd and Pb, with RCs being absent in control fish.	Mediation by stressor-related factors different from cortisol.	Transmission electron microscopy, immunogold labelling and cytochemistry on ultrathin sections from either LX-112 or Spurr’s resin-embedded samples.	Not provided.	[97]
	Cd (4.47 mg L^−1^, 5.63 mg L^−1^, 7.08 mg L^−1^, 8.91 mg L^−1^) for 24 and 48 h of experimental exposure.	*Dicentrarchus labrax*	Increase in the number of RCs (quantitatively assessed) and effect on RCs’ ultrastructure (qualitatively assessed) in both the intestine and the renal tubules.	Not provided.	Light microscopy on sections from paraffin-embedded samples and transmission electron microscopy on ultrathin sections from epoxy resin-embedded samples.	Anova and Ancova test on RCs’ number.	[83]
	Hg (251 µg L^−1^, 355 µg L^−1^, 501 µg L^−1^) for 24 and 48 h of experimental exposure.	*Dicentrarchus labrax*	Increase in the number of RCs (quantitatively assessed) and effect on RCs’ ultrastructure (qualitatively assessed) in both the intestine and the renal tubules.	Not provided.	Light microscopy on sections from paraffin-embedded samples and transmission electron microscopy on ultrathin sections from epoxy resin-embedded samples.	Anova test, Kruskal–Wallis and Jonckheere–Terpstra tests with Monte Carlo exact test extension on RCs’ number.	[82]
	ZnO nanoparticles (20% of the estimated LC_50_, 0.69 mg L^−1^) for 60 days of experimental exposure.	*Danio rerio*	Effect on RCs’ ultrastructure (qualitatively assessed) in olfactory rosettes.	Not provided.	Transmission electron microscopy on ultrathin sections from epoxy resin-embedded samples.	Not provided.	[93]
Organic chemicals	N-nitrosodiethylamine (57 mg L^−1^) for 5–6 weeks of experimental exposure.	*Cyprinodon variegatus*	Increase in the number of RCs and effect on RCs’ ultrastructure (both qualitatively assessed), from 21 to 140 weeks from the experimental start, associated with the hepatic proliferative (reactive, pre-neoplastic and neoplastic) response.	Not provided.	Light microscopy on sections from paraffin-embedded samples and transmission electron microscopy on ultrathin sections from epoxy resin-embedded samples.	Not provided.	[92,142]
	Stam^®^ M-4 (Propanil) (3.16, 6.31 and 12.6 mg L^−1^) for 24, 48 h of experimental exposure.	*Squalius cephalus*	Decrease and increase in the number of RCs (quantitatively assessed) respectively in the heart bulbus arteriosus and in the gills and effect on RCs’ ultrastructure (qualitatively assessed) in the heart, the gills, the intestine, the liver and the kidney.	Physicochemical alteration of cytomembrane and selective inhibition of lysosomal enzymes, resulting in the impaired digestion of phagocytised cytomembranes and myelinsomes formation (with regard to qualitative RCs alterations).	Light microscopy on sections from paraffin-embedded samples and transmission electron microscopy on ultrathin sections from epoxy resin-embedded samples.	Anova test, linear regression and curve-fitting on RCs’ number.	[86]
	Terbuthylazine (3.55 mg L^−1^, 5.01 mg L^−1^, 7.08 mg L^−1^) for 24, 48 h of experimental exposure.	*Dicentrarchus labrax*	Increase in the number of RCs (quantitatively assessed) and effect on RCs’ ultrastructure (qualitatively assessed) in the gills, the intestine and the renal tubules.	Not provided.	Light microscopy on sections from paraffin-embedded samples and transmission electron microscopy on ultrathin sections from epoxy resin-embedded samples.	Anova and Ancova test on RCs’ number.	[85]
	Methyl parathion (4 mg L^−1^ and 8 mg L^−1^) for 10 days of experimental exposure.	*Oreochromis niloticus*	Increase in the number of RCs in the gills (only in fish exposed to 4 mg L^−1^).	Not provided.	Light microscopy on thin sections from glycol methacrylate-embedded samples.	Kruskal–Wallis test with Mann–Whitney (Bonferroni correction) post-hoc test.	[80]
	Perfluorooc- tanoic acid (PFOA) (200 ng L^−1^ and 2 mg L^−1^ PFOA) for 56 days of experimental exposure. PFOA concentration in the kidney of fish exposed to 2 mg L^−1^ PFOA for 56 days = 1.08 ± 0.54 ng g^−1^ wet weight (mean ± standard deviation).	*Cyprinus carpio*	Effect on RCs’ number (numerically quantified) and RCs’ ultrastructure (qualitatively assessed) in the kidney.	RCs acting as alternative sentinel cells responding directly (via TLR) or indirectly (via tissue damage and/or stress-induced response) to PFOA.	Light microscopy on semithin sections and transmission electron microscopy on ultrathin sections from epoxy resin-embedded samples.	Discrete distribution models on RCs’ distribution and Kruskal–Wallis test on the total number of RCs.	[81]
	Perfluorooc- tanoic acid (PFOA) (200 ng L^−1^ and 2 mg L^−1^ PFOA) for 56 days of experimental exposure. PFOA concentration in the kidney of fish exposed to 2 mg L^−1^ PFOA for 56 days = 1.08 ± 0.54 ng g^−1^ wet weight (mean ± standard deviation).	*Cyprinus carpio*	Effect on RCs’ distribution pattern (numerically quantified) and increased RCs’ exocytosis activity (semi-quantitatively assessed) in the renal hematopoietic tissue.	RCs acting as alternative sentinel cells responding directly (via TLR) or indirectly (via tissue damage) to PFOA.	Light microscopy on semithin sections and transmission electron microscopy on ultrathin sections from epoxy resin-embedded samples.	Discrete distribution models on RCs’ distribution and repeated measure Friedman test on RCs’ frequency distribution.	[8]
	Perfluorooc- tanoic acid (PFOA) (200 ng L^−1^ and 2 mg L^−1^ PFOA) for 56 days of experimental exposure.	*Cyprinus carpio*	Increased RCs’ degranulation (numerically quantified) in the hematopoietic tissue.	Not provided.	Light microscopy and image (texture) analysis on ultrathin sections from epoxy resin-embedded samples.	Linear discriminant analysis on RCs’ texture features.	[99]
Miscellaneous chemicals/pollutants	0.3–0.7% chicken manure, polluted water from the river Rhine, up to 30 days of experimental exposure.	*Cyprinus carpio* *Oncorhynchus mykiss*	Appearance of RCs in the skin, with RCs being absent in control fish.	Mediation by stressor-related factors different from cortisol.	Transmission electron microscopy, immunogold labelling and cytochemistry on ultrathin sections from either LX-112 or Spurr’s resin-embedded samples.	Not provided.	[97]
	Dexamethasone-21-isonicotinate (approximately 2 mg kg^−1^ body mass) sampled 6, 24, 48, 72, and 96 h after the intraperitoneal injection.	*Carassius auratus*	Depletion of RCs in the heart bulbar lumen anchored to lining endothelial cells and occurrence of “bleb” discharge modality.	Modified expression of superficial adhesive molecules in RCs and endothelial cells.	Light microscopy on sections from paraffin-embedded samples and on semithin sections from epoxy resin-embedded samples. Transmission electron microscopy on ultrathin sections from epoxy resin-embedded samples.	Anova test on RCs’ number and chi-square test on RCs position with respect to the endothelium and discharge modality.	[101]
	Natural exposure to lake-polluted water/sediments (mining effluents).	*Perca fluviatilis*	Increase in the number of RCs in the gills.	Not provided.	Light microscopy on thin sections from epoxy resin-embedded samples.	Anova test on RCs’ number.	[94]
	Natural exposure to eutrophic, supereutrophic and hypereutrophic water bodies.	*Oreochromis niloticus*	Increase in the number of RCs in the gills of fish from hypereutrophic waters and positive correlation of RCs’ percentage with Trophic State Index.	Not provided.	Light microscopy on thin sections from glycol methacrylate-embedded samples.	Anova test on RCs’ number and Pearson correlation test between RCs’ percentage and Trophic State Index.	[91]
	Natural exposure to an oil spill event (1800 Mg of diesel fuel and 800 Mg of fuel oil).	*Abramis brama*	Increase in the number of RCs (quantitatively assessed) and effect on RCs’ ultrastructure (qualitatively assessed) in the renal collecting ducts.	Not provided.	Light microscopy on sections from paraffin-embedded samples and transmission electron microscopy on ultrathin sections from epoxy resin-embedded samples.	Anova test on RCs’ number.	[87]
	Natural exposure to river-polluted water/sediments (metals).	*Prochilodus argenteus*	Increased occurrence of RCs in the brachial interlamellar spaces of fish from the impacted site, particularly in male fish, compared to the reference site.	Not provided.	Light microscopy on sections from paraffin-embedded samples.	Chi-square test for the comparison of RCs occurrence in the interlamellar spaces according to sampling site and Fisher test for the comparison of RCs occurrence according to gender.	[79]
	Native and polluted (through 3-month deployment in the Milazzo harbour) 0.5 mm diameter PVC pellets, incorporated in feed (0.1% *w*/*w*), up to 90 days of feeding.	*Dicentrarchus labrax*	Increased occurrence of RCs in the intestine of fish fed with native and polluted PVC pellets.	Not provided.	Light microscopy.	Not provided.	[95]
Environmental stressors	Gradual changes in salinity from seawater (34.7‰) either to freshwater (0‰), over 96 h, or to hypertonic seawater (48.4‰) over 7 days of experimental exposure.	*Monodactylus argenteus*	Slight decrease in RCs number and effect on RCs’ ultrastructure in the gills of fish adapted to hypertonic seawater and near absence of RCs in fish adapted to freshwater.	Not provided.	Light microscopy and transmission electron microscopy, respectively, on semithin and ultrathin sections from epoxy resin-embedded samples.	Not provided.	[29]
	Distilled water for 0.5, 1, 2, 4, 7 and 14 days of experimental exposure.	*Perca fluviatilis*	Occurrence of RCs in the interlamellar space of the primary gill filaments after 7 days ongoing.	Not provided.	Light microscopy and transmission electron microscopy, respectively, on semithin and ultrathin sections from epoxy resin-embedded samples.	Not provided.	[34]
	Distilled water, brackish water (5 g sea salt L^−1^), H_2_SO_4_ acidified water (pH 5–6), up to 30 days of experimental exposure, elevated temperature by 7 °C, within 60 min.	*Cyprinus carpio* *Oncorhynchus mykiss*	Appearance of RCs in the skin, particularly in fish exposed to distilled water, with RCs being absent in control fish.	Mediation by stressor-related factors different from cortisol.	Transmission electron microscopy, immunogold labelling and cytochemistry on ultrathin sections from either LX-112 or Spurr’s resin-embedded samples.	Not provided.	[97]
	Calcium-deficient artificial freshwater and NH_4_^+^ (2.5 mM) (in *Cyprinus carpio*) and Cd^2+^ (5 mg L^−1^) (in *Orochromis mossambicus*) as calcium absorption inhibitors for 15, 30, 45, 75 and 90 days of experimental exposure.	*Cyprinus carpio* *Orochromis mossambicus*	Increased occurrence of RCs in the head kidney.	Not provided.	Transmission electron microscopy.	Not provided.	[31]
	Freshwater for 24, 48, and 96 h of experimental exposure.	*Dicentrarchus labrax*	Increase in the number of RCs (quantitatively assessed) and effect on RCs’ ultrastructure (qualitatively assessed) in the gills, the intestine and the renal tubules.	Not provided.	Light microscopy on sections from paraffin-embedded samples and transmission electron microscopy on ultrathin sections from epoxy resin-embedded samples.	Anova test on RCs’ number.	[84]
	After 1 h onwards of experimental wounding.	*Cyprinus carpio* *Oncorhynchus mykiss*	Appearance of RCs in the skin, with RCs being absent in control fish.	Mediation by stressor-related factors different from cortisol.	Transmission electron microscopy, immunogold labelling and cytochemistry on ultrathin sections from either LX-112 or Spurr’s resin-embedded samples.	Not provided.	[97,98]
	Restraint stress (by netting) for 24 h.	*Cyprinus carpio*	Increase in the number of RCs in the kidney.	Alpha-MSH immunoreactivity suggestive of an interaction between RCs’ immune and endocrine regulation.	Light microscopy on sections from paraffin-embedded samples.	Student’s *t*-test on RCs’ number.	[88]
	Overcrowding, with fish housed at two stocking densities (20 kg m^−3^ and 80 kg m^−3^), sampled after 2 and 24 h.	*Dicentrarchus labrax*	Increase in the number of RCs in gills.	Possible mediation of the increased plasma cortisol.	Light microscopy on sections from paraffin-embedded samples, as well as on semithin sections from epoxy resin-embedded samples. Transmission electron microscopy on ultrathin sections from epoxy resin-embedded samples.	Anova test on RCs’ number.	[89]

## Data Availability

No new data were created or analysed in this study. Data sharing is not applicable to this article.
